# Factors Associated with Burnout Syndrome in Serbian Healthcare Workers During the COVID-19 Pandemic

**DOI:** 10.3390/healthcare13020106

**Published:** 2025-01-08

**Authors:** Teodora Safiye, Elvis Mahmutović, Emir Biševac, Velida Zimonjić, Draško Dubljanin, Andreja Kovačević, Nemanja Nenezić, Aleksandar Radlović, Zerina Salihagić, Aldina Ajdinović, Slaviša Minić, Elma Šaćirović, Jovana Uzelac, Zana Dolićanin, Jakša Dubljanin

**Affiliations:** 1 Department of Psychology, State University of Novi Pazar, 36300 Novi Pazar, Serbia; 2 Department of Biomedical Sciences, State University of Novi Pazar, 36300 Novi Pazar, Serbia; ehmahmutovic@np.ac.rs (E.M.); ebisevac@np.ac.rs (E.B.); zsalihagic@np.ac.rs (Z.S.); aajdinovic@np.ac.rs (A.A.); minicvms@gmail.com (S.M.); esacirovic@np.ac.rs (E.Š.); zdolicanin@np.ac.rs (Z.D.); 3 Quality Assurance Office, State University of Novi Pazar, 36300 Novi Pazar, Serbia; vkijevcanin@np.ac.rs; 4 Faculty of Medical Sciences, University of Kragujevac, 34000 Kragujevac, Serbia; draskodubljanin@gmail.com (D.D.); doktorkole86@gmail.com (A.K.); aleksandarradlovic31@gmail.com (A.R.); drjale81@gmail.com (J.D.); 5 Department of Pulmonology, University Clinical Hospital Center Zvezdara, 11120 Belgrade, Serbia; 6 Department of Cardiopulmonary Rehabilitation, Institute for Rehabilitation Belgrade, 11000 Belgrade, Serbia; 7 Department of Medical Studies Ćuprija, Academy of Educational and Medical Vocational Studies Kruševac, 35230 Ćuprija, Serbia; nenezicnemanja@yahoo.com; 8 Faculty of Medicine, University of Novi Sad, 21000 Novi Sad, Serbia; jovana.uzelac@mf.uns.ac.rs; 9 General Hospital Atlas, 11000 Belgrade, Serbia

**Keywords:** burnout syndrome, healthcare workers, COVID-19

## Abstract

**Background/Objectives**: The COVID-19 pandemic imposed immense stress on healthcare systems worldwide, significantly affecting the mental well-being of healthcare workers (HCWs). This study examined the sociodemographic, occupational, and COVID-19-related predictors of burnout syndrome among Serbian HCWs. **Methods**: A cross-sectional survey of 400 doctors and nurses utilized the Maslach Burnout Inventory to assess emotional exhaustion, depersonalization, and personal accomplishment. Sociodemographic, work-related, and COVID-19-related data were collected via a questionnaire. **Results**: Key predictors of emotional exhaustion included female gender, inadequate rest, sleep disturbances, and frontline COVID-19 work. Depersonalization was associated with fewer children, temporary employment, and COVID-19 frontline duties. Conversely, older age, religiosity, larger households, and a higher socioeconomic status positively correlated with personal accomplishment. **Conclusions**: These findings emphasize the urgent need for targeted interventions, including improved working conditions and mental health support, to reduce burnout and enhance HCWs’ well-being during medical crises.

## 1. Introduction

Healthcare workers (HCWs) work in a stressful and demanding environment where their job responsibilities are constantly expanding. The onset of the 2019 coronavirus disease (COVID-19) pandemic, a global health emergency, added stress to the healthcare system, leading to high levels of psychological exhaustion and burnout among HCWs [1]. During the COVID-19 pandemic, healthcare professionals experienced increased work overload, and despite not having precise knowledge of the disease, they confronted a tremendous demand of patients while dealing with limited human resources and inadequate facilities in health services [2].

Burnout is a phenomenon characterized by extreme emotional depletion and poor job adaptation as a result of ongoing occupational stress [3]. Christina Maslach described burnout syndrome in terms of emotional exhaustion (EE), depersonalization (DP), and personal accomplishment (PA) [4]. Emotional exhaustion is manifested in sensations of exhaustion from psychological efforts at work, while depersonalization is a response of detachment, indifference, and unconcern toward the work being carried out and/or the people who receive it. Personal accomplishment is reflected in negative professional self-evaluation and doubts about efficient work performance [5].

Studies have documented the incidence of burnout among healthcare workers (HCWs) worldwide, revealing rates of 45% in the United States, 35% in China, and 40% in Italy during the COVID-19 epidemic. Burnout in many different healthcare systems has repeatedly been found to be predicted by gender, workload, and emotional stress [6]. In Serbia, a country marked by a poor healthcare system, a high patient-to-healthcare worker ratio, and a constant tendency of HCWs to seek better-paying jobs in other countries, the COVID-19 epidemic put great strain on healthcare workers. Limited resources, low salaries, a continuous mismatch between the supply and demand of physicians and nurses, and institutional barriers heightened stress and burnout, underlining the need to look at this problem [7].

The COVID-19 pandemic has made occupational health more important than ever, according to a rising body of scientific research. Healthcare professionals, in particular, have experienced significant psychological exhaustion as a result of the epidemic. Numerous studies on burnout have been conducted worldwide since the COVID-19 epidemic, using samples of frontline healthcare professionals, doctors, nurses, and pharmacists [5,8,9].

Previous studies concerning burnout among healthcare workers investigated a wide range of burnout predictors. These included demographic factors, professional and clinical practice characteristics, and COVID-19-related factors [10]. One study in China found that during the COVID-19 pandemic, over one-third of HCWs experienced severe burnout symptoms, which were independently associated with personal and work-related factors [11]. Other research suggested that factors such as age, socioeconomic status, daily working hours, workload, inadequate protection while working in a hospital, having more years of work experience, working more night shifts, having comorbidities, and having fewer paid vacation days were associated with burnout among doctors and nurses [12,13,14].

### Purpose and Hypotheses

Starting from the theoretical framework and the literature findings, in this study, we aimed to examine the relationship between sociodemographic variables, working conditions, variables related to COVID-19, and burnout syndrome in Serbian healthcare workers during the COVID-19 pandemic. Our hypotheses:

(1) Emotional exhaustion and depersonalization are higher in frontline workers, women, and those with temporary employment.
(2) Personal accomplishment is increased with age, religiosity, and stronger socioeconomic status.
(3) Burnout levels differ across groups based on gender, age, and work experience.

Understanding the unique stressors in this population addresses a critical knowledge gap and also provides insights into burnout trends in other resource-limited healthcare systems. As far as we know, our study was the first conducted in Serbia to examine the role of these specific factors in predicting and explaining burnout syndrome in HCWs during the pandemic.

## 2. Materials and Methods

### 2.1. Study Design, Sample, and Procedures

A cross-sectional, descriptive study was designed to examine factors associated with burnout among HCWs working in Serbia during the COVID-19 pandemic. The studied population included doctors and nurses of both sexes who were actively working with patients in Serbia at the time the study was conducted.

Ethical considerations were addressed, and approval was obtained from the Institutional Review Board of the University of Belgrade, Faculty of Philosophy, Department of Psychology, Serbia (No. 2021-58). The study was conducted in accordance with the ethical guidelines and principles outlined in the Declaration of Helsinki [15]. The consent process included a clear explanation of the purpose of the study, procedures, potential risks, benefits, and voluntariness of participation. Participants were also informed about the confidentiality and anonymity of their responses. Consent was obtained by asking the participants to sign the consent form before proceeding with the survey.

The sample size was calculated at a minimum of 327 subjects, taking into account a population size of 2163, an effect size of 0.3, an alpha error of 0.05, and a study power of 0.95. The sample size was calculated using the Sample Size Calculator from the Australian Bureau of Statistics, an open-source calculator. The population size (N = 2163) represents active HCWs in one of Serbia’s four tertiary hospitals. The relatively small population reflects Serbia’s limited healthcare workforce.

We used a non-probability sampling method, and the focus was on healthcare workers employed in tertiary hospitals dealing with COVID-19 patients directly. Non-probability sampling was chosen due to logistical constraints, including unavailability or limited access to comprehensive databases of healthcare workers and the need to recruit participants directly from their work environment. This approach allowed targeted recruitment of healthcare workers who were actively engaged in patient care during the COVID-19 pandemic, ensuring that the sample reflected those most affected by the crisis.

Inclusion criteria: Age 18–65 years. Active engagement in patient care roles. Signed informed consent to participate in this study. Exclusion criteria: HCWs not directly involved in patient care. 

Voluntary withdrawal from the study was guaranteed, and common method bias was mitigated through anonymous responses and counterbalancing question order.

### 2.2. Instruments

The Maslach Burnout Inventory–Human Services Survey (MBI-HSS), which consists of 22 items, was used to measure the degree of burnout [16]. This questionnaire evaluated three aspects of burnout. Eight items were used to measure personal accomplishment (PA), five items were used to measure depersonalization (DP), and nine questions were used to measure emotional exhaustion (EE). Respondents were asked to rate what they felt on a 7-point Likert-type scale for each of the 22 items, with the range from never experiencing such feelings (value 0) to experiencing them every day (value 6). A high score for PA correlates with a lower level of burnout, whereas high scores for EE and DP correspond to a higher degree of burnout. The scores of each subscale are reported separately and cannot be added up as a total score [16]. In our sample, the subscales of emotional exhaustion, depersonalization, and personal accomplishment had good reliability, and Cronbach’s alpha coefficients were 0.89, 0.71, and 0.74, respectively. All average variance extracted (AVE) values in our sample were >0.50, indicating that consistency between items is acceptable. AVE values from the original scale validation were referenced, ensuring construct validity [16].

For the assessment of sociodemographic variables, working conditions, and variables related to COVID-19, a special questionnaire covering three areas was constructed for the purposes of this research. The first was sociodemographic data: gender (1 = male, 2 = female), age, marital status (1 = married or cohabiting, 2 = single), number of children, socioeconomic status (from 1 = very poor to 5 = excellent), number of years of service, the number of people with whom the respondent lives, degree of religiosity (from 1 = not religious at all to 5 = very religious), and health condition (from 1 = excellent to 5 = very bad). The second part of the questionnaire consisted of work-related variables: type of occupation (1 = general practitioners and specialist doctors, 2 = medical technicians and nurses), current work status (1 = employee on a full-time permanent basis, 2 = temporary employee, intern, or volunteer), work environment (1 = COVID-19 zone, 2 = regular clinical conditions), contact with contaminated material during work (1 = yes, 2 = no), job dislocation (1 = work in the registry office, 2 = displacement due to the pandemic), shift work (1 = day shifts only, 2 = shifts that include night work and on-call), and enough time to rest after work (1 = yes, 2 = no). The third part of the questionnaire consisted of variables related to COVID-19: isolation, hospitalization of the respondent due to coronavirus infection, vaccination, concern that a family member and/or someone living with them may be infected, belonging to a risk group due to age or chronic disease, smoking, increased use of alcohol, increased use of anxiolytics, and sleep problems. The respondent could answer the questions by choosing the number 1 = yes or 2 = no.

### 2.3. Data Analyses

Statistical analysis was performed using licensed SPSS for Windows, version 22.0 of the Statistical Package for the Social Sciences (IBM Corp., Armonk, NY, USA). Statistical processing and data analysis included methods of descriptive inference statistics (frequencies, percentages, arithmetic means, standard deviations, minimum and maximum values, skewness, and kurtosis). Cronbach’s alpha was used as a measure of internal consistency to check the reliability of the subscales of the MBI-HSS instrument. The statistical significance level was set at *p* < 0.05 (two-tailed).

Rationale for Correlation and Regression Analysis: Correlation analysis was initially performed to explore bivariate relationships between predictors and the three dimensions of burnout. This exploratory step was necessary, and it allowed the identification of significant predictors, which were then included in subsequent multiple hierarchical regression analyses. Regression analysis was used to determine the extent to which multiple variables collectively predicted emotional exhaustion, depersonalization, and personal accomplishment.

Assumptions for Regression Analysis: Linearity and homoscedasticity of residuals were verified using scatterplots. Multicollinearity was assessed using variance inflation factor (VIF) values, all of which were below 5. Durbin–Watson statistics confirmed no autocorrelation (values between 1.5 and 2.5). Categorical predictors (e.g., gender and work status) were appropriately coded as dummy variables.

### 2.4. Recruitment Process

Participants were gathered directly through direct involvement in cooperation with the University Clinical Center of Kragujevac. Doctors and nurses received self-administered questionnaires from the study team during working hours. Respondents gave written informed consent before answering the survey; participation was voluntary. Data collection ran from 1 July 2021 to 1 February 2022.

The response rate was sufficient for statistical analysis; hence, selection bias still exists even in this case. Those most affected may have opted out of participation due to extreme burnout, therefore underrepresenting themselves. Furthermore, hiring participants from a single major hospital could limit the generalizability of results to rural or primary care professionals. More inclusive sampling strategies should be used in the next studies to reduce this bias.

## 3. Results

The questionnaire was filled out by 514 respondents, but after eliminating those who did not meet all the criteria for inclusion in the study, inadequate answers, and outliers, 400 respondents were included in the final analysis.

### 3.1. Sociodemographic Characteristics of the Examined Sample

The research sample consisted of 400 respondents, 136 men (34.0%) and 264 (66.0%) women, aged 19 to 62. The average age of the respondents was 40.16 years (SD = 9.45); the median was 40 years of age. A total of 286 (71.5%) respondents stated that they live in a marriage or cohabitation, while 114 (28.5%) of them were single. More detailed information about the sociodemographic characteristics of respondents is shown in Table 1.

### 3.2. Work-Related Characteristics of the Examined Sample

In terms of occupation, the sample included 140 general practitioners and specialist doctors (35%) and 260 medical technicians and nurses (65%). The average length of service of the respondents was 15.36 years (SD = 10.42). A total of 137 (34.3%) respondents answered that they have enough time to rest after work, while 263 (65.8%) answered that they do not. Table 2 provides more detailed information about the investigated work-related characteristics of the respondents.

### 3.3. COVID-19-Related Characteristics of the Examined Sample

In this part of the survey, a series of questions related to the COVID-19 pandemic were asked, to which respondents answered yes (value 1) or no (value 2). The COVID-19-related characteristics of the respondents are shown in Table 3.

### 3.4. Correlations of Sociodemographic, Work-Related, and COVID-19-Related Characteristics with Burnout Syndrome Dimensions

Correlations between the sociodemographic characteristics of the respondents and the dimensions of burnout syndrome are shown in Table 4.

Emotional exhaustion was significantly positively related to gender (r = 0.38, *p* < 0.01), while it was significantly negatively related to socioeconomic status (r = −0.19, *p* < 0.01) and self-assessment of health condition (r = −0.21, *p* < 0.01). The findings indicated that emotional exhaustion was greater in women and that emotional exhaustion increased the lower the socioeconomic status of the respondents and the worse the health condition.

Depersonalization was significantly positively related to gender (r = 0.18, *p* < 0.01) and negatively related to the age of the respondents (r = −0.11, *p* < 0.01) and the number of children (r = −0.17, *p* < 0.01). The findings indicated that with the female gender come more experiences of depersonalization and that depersonalization decreased with the increase in the age of the respondents and with the increase in the number of their children.

Personal accomplishment was significantly positively related to the age of the respondents (r = 0.17, *p* < 0.01), the number of children (r = 0.22, *p* < 0.01), the number of household members (r = 0.21, *p* < 0.01), and the religiosity of the respondents (r = 0.14, *p* < 0.01). The findings indicated that the experience of personal accomplishment increased with the age of the respondents, with the number of their children, with the growth in the number of their households, and with the increase in their religiosity. Also, with the decreasing age of the respondents, the decreasing number of children they have, the decreasing number of their household members, and the reduction in their religiosity, the perception of their personal accomplishment decreased (Table 4).

Table 5 shows the correlations between working conditions and dimensions of burnout syndrome.

Emotional exhaustion was most significantly related to the answer to the question, “Do you have enough time to rest after work?” (r = 0.38, *p* < 0.01). The correlation with the work environment was most unfavorable (r = −0.31, *p* < 0.01), followed by the respondent’s interaction with contaminated materials during work (r = −0.22, *p* < 0, 01), and then working status (r = −0.15, *p* < 0.01). These findings indicated that emotional exhaustion was greater when respondents estimated that they did not have enough time to rest after work when they worked in a COVID-19 zone, came into contact with contaminated material, and were employed on a full-time, permanent basis (i.e., for an indefinite period of time). Emotional exhaustion was positively related to the occupation (r = 0.15, *p* < 0.01), which indicated that medical technicians experienced greater exhaustion than doctors. Job dislocation (r = 0.13, *p* < 0.01) indicated that exhaustion was greater in respondents who were transferred to other departments due to the pandemic, as well as those who carried out shift work (r = 0.10, *p* < 0.05), which indicated that respondents who work in addition to day and night shifts experienced greater exhaustion than those who only work day shifts.

Depersonalization was significantly negatively related to the mentioned work environment variable (r = −0.26, *p* < 0.01) and then to work status (r = −0.13, *p* < 0.01), while it was positively related to lack of time for rest (r = 0.13, *p* < 0.01) and work that includes night shifts (r = 0.13, *p* < 0.01). These findings indicated that depersonalization was greater in subjects who worked in a COVID-19 zone compared to subjects who worked in regular clinical conditions, that depersonalization was greater in employees on a full-time permanent basis, that depersonalization in subjects increased with the strengthening of the feeling that they lack time for rest after work, and that respondents who also work night shifts have more depersonalization than respondents who only work day shifts.

The experience of personal accomplishment was significantly positively related to the number of years of service (r = 0.16, *p* < 0.01). The findings indicated that with increasing length of service among respondents, their experience of personal accomplishment strengthens (Table 5).

Table 6 shows the correlations between the characteristics associated with COVID-19 and burnout syndrome dimensions.

Emotional exhaustion had a significant negative correlation with the variable operationalized by a question related to the existence of concern that the respondent will infect his or her household with coronavirus (r = −0.32, *p* < 0.01), then with a question related to difficulties with sleep (r = −0.35, *p* < 0.01), as well as with the question of increasing the use of anxiolytics (r = −0.20, *p* < 0.01), and with the question related to the increase in cigarette consumption (r = −0.10, *p* < 0.05). It was also negatively related to the question of whether the respondent belongs to a risk group due to age or chronic disease (r = −0.16, *p* < 0.01), as well as whether the respondent has been vaccinated (r = −0.11, *p* < 0.05). The findings showed that the increase in emotional exhaustion in the respondents occurred together with an increase in their concern that they would infect their household, then with the increase in their sleeping difficulties, with the increase in the use of anxiolytics, and in the case of smokers, with the inability to increase the consumption of cigarettes. Furthermore, emotional exhaustion was higher in respondents who belong to the risk group due to age or chronic illness, as well as in those who have not been vaccinated.

Depersonalization had a significant negative correlation with the variable that was operationalized by a question related to the existence of respondents’ concern that their household will be infected with coronavirus (r = −0.16, *p* < 0.01), then with a question related to difficulties with sleep (r = −0.13, *p* < 0.01), and also with the question related to the increase in cigarette consumption (r = −0.10, *p* < 0.05). These findings indicate that the growth of depersonalization in the respondents occurred together with the increase in their concern that they will infect their household, with the occurrence of sleeping difficulties, and in smokers who increased cigarette consumption.

There were no significant correlations between variables related to COVID-19 and personal accomplishment as a dimension of burnout syndrome (Table 6).

### 3.5. Regression Analyses of the Dimensions of Burnout Syndrome Based on All Examined Variables

The following three tables (Table 7, Table 8 and Table 9) show the results of the multiple hierarchical regression analysis of the dimensions of burnout based on all the characteristics examined in this research (stepwise method). In each regression analysis, only statistically significant predictors are presented in order according to the significance of the t-statistic (*p*-value). Multicollinearity was assessed using variance inflation factor (VIF) tests, and no problems were indicated, as the values for VIF in all three regression models of burnout dimensions were less than 5. Also, the values of Durbin–Watson statistics in all three regression models were within the limits of 1.5 to 2.5, which indicates that there were no autocorrelation problems.

Table 7 shows the results of the regression analysis of emotional exhaustion as a dimension of burnout syndrome. Seven significant predictors of emotional exhaustion were singled out. The regression model explains 37% of the variance in emotional exhaustion (adj. R^2^ = 0.37, *p* < 0.05).

The gender of the subject was the most significant predictor of emotional exhaustion as a dimension of burnout syndrome (ß = 0.27, *p* < 0.01). The findings indicate that women had more emotional exhaustion as a dimension of burnout. The second significant predictor for explaining the variance in emotional exhaustion was the answer to the specific question of whether the respondent had enough time to rest after work. When respondents estimated that they did not have enough time to rest after work, their emotional exhaustion was higher (ß = 0.23, *p* < 0.01). The respondent’s answer to the question: Have you had problems sleeping since the beginning of the epidemic? (1. Yes, or 2. No) was the next most significant predictor for explaining the variance in emotional exhaustion (ß = −0.17, *p* < 0.01). The findings indicated that respondents who had sleep problems during the pandemic experienced more emotional exhaustion. The work environment was a significant predictor of emotional exhaustion as a dimension of burnout (ß = −0.14, *p* < 0.01). Emotional exhaustion was lower if the respondents worked in regular clinical conditions and not in the COVID-19 zone. Working in the COVID-19 zone predicted greater emotional exhaustion. The subject’s health condition was a significant predictor of emotional exhaustion as a dimension of burnout (ß = −0.12, *p* < 0.01). The findings indicated that, with a lower assessment of the health status of the subjects, their emotional exhaustion was greater. The answer to the question, “Are you worried about infecting your household with the coronavirus?” (1. Yes, or 2. No) in this model represented a significant predictor of emotional exhaustion as a dimension of burnout at work (ß = −0.11, *p* < 0.01). The findings indicated that respondents who were worried about infecting their household experienced more emotional exhaustion. Work status was also a significant predictor of emotional exhaustion (ß = −0.14, *p* < 0.05). The findings indicated that respondents who have a permanent job contract had less emotional exhaustion at work compared to employees who do not have a permanent job (temporary employees, interns, or volunteers) (Table 7).

Table 8 shows the regression analysis of depersonalization as a dimension of burnout syndrome, which resulted in a model with five statistically significant predictors. The depersonalization regression model explains approximately 14% of its variance (adj. R^2^ = 0.14, *p* < 0.05).

In this model, the number of children the respondents have was a significant predictor of depersonalization as a dimension of burnout syndrome, which explained most of its variance in the model (ß = −0.25, *p* < 0.01). The findings indicated that the more children the respondents had, the weaker their experience of depersonalization at work. The work environment was the next significant predictor of depersonalization as a dimension of burnout (ß = −0.18, *p* < 0.01). Depersonalization was less if the respondents worked in regular clinical conditions and not in a COVID-19 zone. Working in a COVID-19 zone predicted greater depersonalization. Gender was the next significant predictor of depersonalization as a dimension of burnout syndrome (ß = 0.16, *p* < 0.01). The findings indicated that the experience of depersonalization was more pronounced in female respondents. Work status was a significant predictor of depersonalization as a dimension of burnout (ß = −0.13, *p* < 0.01). The findings indicated that the respondents who had a permanent job (i.e., a permanent contract) experienced less depersonalization compared to employees who did not have a permanent job (temporary employees, interns, or volunteers). As with the dimension of emotional exhaustion, respondents who were worried about infecting their household members with coronavirus experienced more depersonalization (ß = −0.09, *p* < 0.05) (Table 8).

Table 9 shows the results of the regression analysis of personal accomplishment as a dimension of burnout syndrome. Five significant predictors of personal accomplishment were singled out. The regression model explains 10% of the variance in personal accomplishment (adj. R^2^ = 0.10, *p* < 0.05). While the R^2^ value for personal accomplishment (Table 9) was low (0.10), this model provides exploratory insights into predictors of this burnout dimension. However, these findings should be interpreted cautiously and verified through further research.

The age of the respondent was the first significant predictor of personal accomplishment as a dimension of burnout syndrome, and in the given model, it explained most of its variance (ß = 0.21, *p* < 0.01). The older the respondents, the more favorable their experience of personal accomplishment at work was. The number of household members with whom the respondents live was a significant predictor of personal accomplishment (ß = 0.18, *p* < 0.01). The findings indicated that with the increase in the number of household members, the respondents’ perception of their personal efficiency at work also increased. The answer to the question “How would you rate your level of religiosity on a scale of 1 to 5”, where the number 1 means “I am not religious at all” and the number 5 means “I am very religious”, was a significant predictor of personal accomplishment (ß = 0.12, *p* < 0.01). The findings indicated that with an increase in the degree of religiosity, the respondents’ experience of personal accomplishment increased. The findings also indicated the following: with weaker religiosity, the respondents’ experience of personal efficiency at work also weakened. Socioeconomic status was a significant predictor of personal accomplishment (ß = 0.11, *p* < 0.01). The findings indicated that with higher socioeconomic status, the experience of personal accomplishment also increased. The reverse is also true: the lower the socioeconomic status of the respondents, the weaker their perception of personal accomplishment. The work status of the respondents was a significant predictor of personal accomplishment (ß = 0.11, *p* < 0.05). The findings indicated that respondents who do not have a permanent job had a more favorable experience of personal accomplishment than respondents who have a permanent job (Table 9).

To facilitate understanding, the most relevant correlations between MBI dimensions and sociodemographic/work-related/COVID-19-related factors are summarized below:

Emotional Exhaustion:

- Significantly associated with female gender (β = 0.27, *p* < 0.01), lack of time to rest (β = 0.23, *p* < 0.01), and sleep problems (β = −0.17, *p* < 0.01). HCWs working in COVID-19 frontline zones (β = −0.14, *p* < 0.01) reported higher emotional exhaustion.

Depersonalization:

- Higher among HCWs with fewer children (β = −0.20, *p* < 0.01) and those working in COVID-19 frontline zones (β = −0.18, *p* < 0.01). Significantly associated with temporary employment (β = −0.13, *p* < 0.01).

Personal Accomplishment:

- Positively associated with older age (β = 0.21, *p* < 0.01), number of household members (β = 0.18, *p* < 0.01), and religiosity (β = 0.12, *p* < 0.01).

### 3.6. Severity of Burnout Dimensions and Group Comparisons

Burnout severity was assessed using the MBI. Mean scores for burnout dimensions were:

Emotional exhaustion: M = 36.03, SD = 11.51.

Depersonalization: M = 8.92, SD = 6.40.

Personal accomplishment: M = 37.45, SD = 6.32.

Gender Differences:

Female HCWs reported significantly higher emotional exhaustion (M = 39.19, SD = 9.89) than males (M = 29.88, SD = 11.98; *p* < 0.05).

Age and Experience Comparisons:

Younger HCWs (≤30 years) experienced higher emotional exhaustion (M = 35.44, SD = 10.50) compared to older workers (>30 years; M = 36.16, SD = 11.73; *p* < 0.05).

There was no significant difference in years of experience when it comes to depersonalization and emotional exhaustion. Less experienced HCWs (<10 years) reported similar depersonalization and exhaustion to those HCWs with >10 years of service.

## 4. Discussion

This research aimed to analyze the association of burnout syndrome in healthcare workers with working conditions, sociodemographics, and COVID-19 characteristics. Our findings confirmed that certain sociodemographic, work-related, and COVID-19-related variables were significant predictors of burnout among healthcare workers.

### 4.1. Sociodemographic Factors

Our research indicates that female healthcare workers (HCWs) encountered markedly elevated levels of emotional exhaustion and depersonalization throughout the COVID-19 epidemic. These results correspond with similar research undertaken in China and Italy, where women had heightened emotional distress [17,18]. These results might point to the two-fold stress experienced by female healthcare professionals who constantly deal with family and caring responsibilities and, at the same time, deal with professional obligations. On the other hand, studies in Turkey and China revealed higher burnout syndromes in men [19,20]. Such differences in study results could arise from cultural variances, professional settings, or distinct social expectations put on male and female medical practitioners, especially in different countries.

Age has an influence on personal performance since older medical professionals express more professional satisfaction, and this trend is consistent with earlier studies showing that age and experience can increase resilience and provide healthcare professionals with the emotional tools required to manage stress [21]. Younger workers might, on the other hand, lack the necessary experience to effectively control pandemic-related stress, which would lead to higher burnout. Similarly, healthcare professionals with more children or bigger households had reduced degrees of depersonalization, presumably due to family support that helped lessen occupational stress [22,23].

Findings from the literature indicate that the younger age of healthcare workers increased the level of burnout during the COVID-19 pandemic, which is in line with our findings [13] since the regression analysis showed that older healthcare workers have a more favorable experience of personal efficiency at work as a dimension of burnout, while age was not a significant predictor of the remaining two dimensions of burnout.

Sociodemographic factors such as the number of children, number of household members, and socioeconomic status influenced burnout among healthcare workers in our research as well. Specifically, our findings indicate that the experience of personal efficacy increases with the age of the respondent, with an increase in the number of household members with whom the respondent lives, with a higher socioeconomic status, and with an increase in their religiosity, while with a larger number of children, the level of depersonalization as a dimension of burnout decreases. The obtained findings are in accordance with previously conducted research that found that with an increase in the number of children, healthcare workers have a lower degree of burnout [24,25]. According to many studies, single workers have a higher level of burnout at work, so it is expected that those who live alone or with a smaller number of household members experience burnout more often, i.e., feel less efficient at work [26,27].

Our findings showed that the higher socioeconomic status of healthcare workers increased personal accomplishment, which is the only one of the three dimensions of burnout whose score is inversely proportional to burnout, which is consistent with previous studies suggesting that higher income reduces burnout [28,29].

An interesting finding is that in the regression model of personal accomplishment as a dimension of burnout, religiosity appears as the most significant predictor, i.e., increasing the degree of religiosity also increases the experience of personal efficiency at work among healthcare workers. This finding is consistent with the results of earlier research that showed that religiosity and spiritual well-being reduce the degree of burnout [30,31,32]. In fact, most studies revealed that spiritual and religious beliefs were correlated with lower levels of burnout [33] and provided evidence for a significant linear negative relationship between religiosity and burnout syndrome in HCWs [34]. 

### 4.2. Working Conditions

Working conditions were important predictors of burnout as healthcare professionals in COVID-19 frontline settings encountered heightened emotional exhaustion and depersonalization compared to their peers in conventional clinical situations. This is not unexpected given the considerable strain experienced by frontline personnel, including prolonged contact with COVID-19 patients, few resources, and the ongoing threat of potentially serious infection. These findings are in line with international research studies and demonstrate similar psychological patterns in different countries, such as Brazil, Japan, and Saudi Arabia [35,36,37].

The inadequate time for rest after shifts significantly exacerbated emotional exhaustion. Insufficient rest periods are acknowledged as a contributing factor to burnout, as they impede healthcare professionals’ mental and physical recovery [10,38]. This highlights the urgent need for healthcare institutions to implement policies that provide adequate resting periods for their workers, particularly in crises. Sleep disorders were a substantial predictor of emotional exhaustion, a finding supported by studies conducted in France and the United States [39,40]. Implementing workplace measures to mitigate sleep issues, such as promoting work–life balance and providing mental health services, may reduce burnout among healthcare professionals.

An interesting paradox emerged regarding employment status, as temporary employment was linked to heightened emotional exhaustion and depersonalization, but at the same time, it was also associated with improved personal achievement. This may be related to the increased incentive among temporary employees to prove themselves in seeking permanent positions, a phenomenon consistent with research on self-efficacy and work engagement [41,42]. Job insecurity remains a significant stressor and must be addressed to alleviate burnout levels.

In our study, health condition was a significant predictor of burnout in the sense that with a lower assessment of the health status of healthcare workers, their emotional exhaustion was higher, which is in agreement with other research conducted around the world [43,44,45].

### 4.3. Relationship with COVID-19

The problems encountered by healthcare workers specific to the pandemic are significant. A considerable percentage of respondents expressed concerns regarding the potential transmission of infection to their household members, which was a major predictor of both emotional exhaustion and depersonalization. This fear is extensively reported worldwide and signifies a distinct psychological burden associated with people working in infectious disease environments [46,47].

Furthermore, the exacerbation of sleep deprivation, along with heightened consumption of anxiolytics, was a definitive sign of the pandemic’s impact on mental health. These findings align with research indicating a significant correlation among sleep problems, pharmaceutical usage, and burnout [40,48]. Facing and solving these problems requires a comprehensive strategy that integrates mental health support with pragmatic solutions, including better protection measures and task management, to mitigate anxieties and diminish stress.

The obtained results confirmed that in our research, emotional exhaustion as a key dimension of burnout was higher in healthcare workers who have sleep problems, which is in full agreement with the studies conducted by Shechter et al. [39] and Coelho et al. [40].

Research conducted during the pandemic showed that one of the biggest concerns of healthcare workers was the possibility of infecting others, especially family members [46]. A significant predictor of burnout, i.e., the dimensions of emotional exhaustion and depersonalization, in our study was the respondents’ concern that they would infect their household members with coronavirus, which is in line with the results of other studies that have shown that healthcare workers had additional concerns related to the COVID-19 pandemic, such as a lack of personal protective equipment and fear of exposure to SARS-CoV-2 at work and taking the virus home to close family members [47], which contributes to burnout [49].

Working at the forefront of the COVID-19 healthcare crisis has resulted in a series of stressful experiences that have an impact on the well-being of healthcare workers. COVID-19-frontline HCWs are at risk for poor mental health and burnout syndrome [35]. In our study, it was shown that working in a COVID-19 zone means a higher level of burnout syndrome, that is, more emotional exhaustion and more depersonalization, compared to working in regular clinical conditions that do not include contact with COVID-19-positive patients. This was quite expected based on numerous previously conducted studies around the world [35,36,37,50,51].

### 4.4. Limitations and Future Research

Despite its strengths, this study also has limitations. The first one is a cross-sectional study design. It is well known that this particular study design restricts our capacity to assess causality. Also, the recruiting method may have introduced a selection bias since severely burned-out healthcare workers may have chosen not to participate, and since this study was conducted in one large healthcare center, the results cannot be properly generalized. Therefore, additional studies are required, especially longitudinal, to assess the causality and document variations in burnout over time and, at the same time, incorporate healthcare workers from a range of healthcare environments, encompassing primary care and rural hospitals. Furthermore, conducting interventional research centered on mental health initiatives, relaxation techniques, and job stability may yield practical insights for mitigating burnout.

The low R^2^ value (0.10) for the personal accomplishment model suggests that additional predictors should be explored in future research. Our findings are also limited by the difference in percentages between male and female HCWs who participated in this study. Despite this, our findings highlight key sociodemographic, COVID-19-related, and occupational factors that influence burnout, which can help in planning psychological support strategies. Furthermore, effective administrative control is important to establish policies and mechanisms to identify and report symptoms of burnout among healthcare workers to promote their well-being [1].

Burnout appears to have a negative effect on all aspects of health, and recognizing the signs of burnout is crucial to mitigating its effects on health [52]. The identification of factors impacting burnout syndrome among healthcare professionals might assist in focusing on specific points for intervention to establish an effective healthcare workforce, particularly in light of the COVID-19 pandemic’s medical crisis. While this research may help in certain policy-specific areas where interventions are needed, more extensive longitudinal and interventional studies will be necessary to determine the best options and assess their efficacy.

## 5. Conclusions

This study offers an in-depth analysis of burnout among Serbian healthcare professionals during the COVID-19 pandemic, pinpointing significant predictors across sociodemographic, occupational, and COVID-19-related characteristics. The female gender, insufficient rest after prolonged shifts, sleep disturbances, frontline work in COVID-19 zones, and job instability substantially led to emotional exhaustion and depersonalization. Conversely, older age, more household members, and religiosity correlated with enhanced personal achievement.

Our findings correspond with international studies while emphasizing the distinct problems encountered by healthcare workers in resource-constrained systems, such as Serbia. Healthcare institutions in charge should prioritize strategies that mitigate the underlying causes of burnout, including the implementation of required and adequate rest intervals, mental health support programs, and job insecurities. Better working conditions for healthcare workers are essential for their welfare and crucial for sustaining an efficient healthcare staff, especially during medical crises and health emergencies, such as the COVID-19 pandemic.

This study highlights several practical implications for improving the well-being of healthcare workers in Serbia. These can also be implemented in other, similar resource-limited countries. Healthcare institutions should prioritize the implementation of mandatory rest periods. Rest periods and recovery time for HCWs, particularly those working in high-stress environments such as red zones of COVID-19 wards, must be adequately organized. Implementing mental health support programs could reduce emotional exhaustion and improve resilience. All HCWs should have access to psychological counseling and stress management training. And this could also be made mandatory for all healthcare workers. When it comes to temporary workers and temporary employment, introducing policies that provide more stable employment conditions could reduce job insecurity and mitigate this type of stress. Finally, encouraging social support and recognizing personal accomplishments in work settings can improve the motivation and morale of healthcare workers, ultimately improving patient care quality.

Subsequent studies should expand upon these findings by investigating longitudinal patterns and assessing the effects of targeted interventions. By proactively reducing burnout, healthcare organizations can increase support for their personnel and guarantee the quality of patient care during crises.

## Figures and Tables

**Table 1:** Sociodemographic characteristics of the sample (N = 400).

Variable		*N* (%)
Gender	Male	136 (34.0)
	Female	264 (66.0)
Age [mean (SD)]		40.16 (9.45)
Marital status	Married/cohabiting	286 (71.5)
	Unmarried/single	114 (28.5)
Number of children	0	119 (29.8)
	1	110 (27.5)
	2≥	171 (42.8)
Number of household members [mean (SD)]		2.77 (1.43)
Socioeconomic status	Very poor	12 (3.0)
	Poor	71 (17.8)
	Good	227 (56.8)
	Very good	76 (19.0)
	Excellent	14 (3.5)
Level of religiosity	I am not religious at all	23 (5.8)
	I am not religious	50 (12.5)
	I have no opinion	109 (27.3)
	I am religious	150 (37.5)
	I am very religious	68 (17.0)
Health condition	Very bad	5 (1.3)
	Bad	23 (5.8)
	Good	123 (30.8)
	Very good	185 (46.3)
	Excellent	64 (16.0)

**Table 2:** Work-related characteristics of the sample (N = 400).

Variable		*N* (%)
Occupation	General practitioners and specialist doctors	140 (35.0)
	Medical technicians and nurses	260 (65.0)
Work environment	COVID-19 zone	200 (50.0)
	Regular clinical settings	200 (50.0)
Number of years of service [mean (SD)]		15.36 (10.42)
Work status	Employee on a full-time permanent basis	357 (89.3)
	Temporary employees/interns/volunteers	43 (10.8)
Work shifts	Day shifts only	185 (46.3)
	Shifts that encompass night work and on-call	215 (53.8)
Contact with contaminated material in the course of work	Yes	337 (84.3)
	No	63 (15.8)
Job dislocation	Displacement due to the pandemic	124 (31.0)
	Work in the registry office	276 (69.0)
Enough time to rest after work	Yes	137 (34.3)
	No	263 (65.8)

**Table 3:** COVID-19-related characteristics of the sample (N = 400).

Question		*N* (%)
Are you worried that you will infect one of the people you live with with coronavirus?	Yes	297 (74.3)
	No	103 (25.8)
Do you belong to a risk group due to age or chronic disease?	Yes	120 (30.0)
	No	280 (70.0)
Were you in isolation due to an infection with coronavirus?	Yes	198 (49.5)
	No	202 (50.5)
Have you been hospitalized due to an infection with coronavirus?	Yes	23 (5.8)
	No	377 (94.3)
Have you received a vaccine against coronavirus?	Yes	307 (76.8)
	No	93 (23.3)
Are you a smoker?	Yes	204 (51.0)
	No	196 (49.0)
If you are a smoker, have you increased your use of cigarettes?	Yes	92 (23.0)
	No (or non-smokers)	308 (77.0)
Have you started consuming and/or increased your consumption of alcohol since the beginning of the pandemic?	Yes	33 (8.3)
	No	367 (91.8)
Have you started using and/or increased the use of anxiolytics since the beginning of the pandemic?	Yes	86 (21.5)
	No	314 (78.5)
Do you have problems sleeping since the beginning of the pandemic?	Yes	187 (46.8)
	No	213 (53.3)

**Table 4:** Correlations of sociodemographic characteristics and dimensions of burnout syndrome.

	**Gender**	**Age**	**Marital Status**	**Number of Children**	**Number of Household Members**	**Socioeconomic Status**	**Religiosity**	**Health Condition**
Age	0.02							
Marital status	−0.06	−0.18 **						
Number of children	0.12 *	0.46 **	−0.47 **					
Number of household members	0.15 **	0.02	−0.33 **	0.49 **				
Socioeconomic status	−0.12 *	−0.10 *	0.01	−0.05	0.02			
Religiosity	0.06	−0.03	−0.05	0.03	0.13 **	0.09		
Health condition	−0.04	−0.14 **	0.11 *	−0.11 *	−0.02	0.20 **	−0.01	
Emotional exhaustion	0.38 **	0.01	−0.06	0.01	0.02	−0.19 **	0.04	−0.21 **
Depersonalization	0.18 **	−0.11 *	0.09	−0.17 **	−0.06	−0.05	−0.00	−0.02
Personal accomplishment	0.03	0.17 **	−0.07	0.22 **	0.21 **	0.08	0.14 **	0.07

Note: ** *p* < 0.01, * *p* < 0.05.

**Table 5:** Correlations of work-related characteristics and dimensions of burnout syndrome.

	Work Status	Years of Service	Occupation	Work Environment	Work Shifts	Contaminated Material	Job Dislocation	Time for Rest
Years of service	−0.30 **							
Occupation	0.00	0.19 **						
Work environment	0.12 *	0.06	−0.07					
Work shifts	0.07	−0.25 **	0.03	−0.20 **				
Contaminated material	0.04	0.03	−0.25 **	0.28 **	−0.23 **			
Job dislocation	−0.07	−0.01	0.06	−0.30 **	0.12 *	−0.14 **		
Enough time to rest after work	0.04	−0.13 *	0.11 *	−0.21 **	0.17 **	−0.09	0.10 *	
Emotional exhaustion	−0.15 **	0.04	0.15 **	−0.31 **	0.10 *	−0.22 **	0.13 **	0.38 **
Depersonalization	−0.13 **	−0.08	0.06	−0.26 **	0.13 **	−0.05	0.08	0.13 **
Personal accomplishment	0.04	0.16 **	0.04	0.03	−0.06	0.02	0.02	0.01

Note: ** *p* < 0.01, * *p* < 0.05.

**Table 6:** Correlations of COVID-19-related variables and dimensions of burnout syndrome.

	Emotional Exhaustion	Depersonalization	Personal Accomplishment
Do you belong to a risk group due to age or chronic disease?	−0.16 **	−0.03	−0.00
Are you worried that you will infect one of the people you live with with coronavirus?	−0.32 **	−0.16 **	0.01
Were you in isolation due to an infection with coronavirus?	0.00	−0.00	0.05
Have you been hospitalized due to an infection with coronavirus?	−0.04	−0.06	0.03
Have you received a vaccine against coronavirus?	−0.11 *	−0.03	0.01
Are you a smoker?	−0.00	0.00	0.00
If you are a smoker, have you increased your use of cigarettes?	−0.10 *	−0.10 *	0.04
Have you started consuming and/or increased your consumption of alcohol since the beginning of the pandemic?	−0.05	−0.01	0.08
Have you started using and/or increased the use of anxiolytics since the beginning of the pandemic?	−0.20 **	−0.07	0.07
Do you have problems sleeping since the beginning of the pandemic?	−0.35 **	−0.13 **	−0.00

Note: ** *p* < 0.01, * *p* < 0.05.

**Table 7:** Regression model of emotional exhaustion.

Outcome Variable: Emotional Exhaustion			
Predictors	ß	t	VIF
Gender	0.27 **	6.58	1.07
Enough time to rest after work	0.23 **	5.37	1.18
Sleep problems	−0.17 **	−4.03	1.18
Work environment (COVID-19 or regular conditions)	−0.14 **	−3.41	1.10
Health condition	−0.12 **	−3.10	1.05
Concern that you will infect the household	−0.11 **	−2.63	1.20
Work status	−0.08 *	−2.10	1.04
R^2^	0.38		
adj. R^2^	0.37		
F Ch.	4.44 *		
Durbin–Watson	2.01		

Note: ** *p* < 0.01, * *p* < 0.05.

**Table 8:** Regression model of depersonalization.

Outcome Variable: Depersonalization			
Predictors	ß	t	VIF
Number of children	−0.20 **	−4.32	1.06
Work environment (COVID-19 or regular conditions)	−0.18 **	−3.81	1.08
Gender	0.16 **	3.41	1.06
Work status	−0.13 **	−2.72	1.07
Concern that you will infect the household	−0.09 *	−2.09	1.04
R^2^	0.15		
adj. R^2^	0.14		
F Ch.	4.38 *		
Durbin–Watson	2.11		

Note: ** *p* < 0.01, * *p* < 0.05.

**Table 9:** Regression model of personal accomplishment.

Outcome Variable: Personal Accomplishment			
Predictors	ß	t	VIF
Age	0.21 **	4.34	1.09
Number of household members	0.18 **	3.82	1.01
Religiosity	0.12 **	2.59	1.03
Socioeconomic status	0.11 **	2.35	1.02
Work status	0.11 *	2.23	1.08
R^2^	0.11		
adj. R^2^	0.10		
F Ch.	4.99 *		
Durbin–Watson	1.90		

Note: ** *p* < 0.01, * *p* < 0.05.

## Data Availability

The data supporting this study’s findings are available from the corresponding author upon reasonable request.
